# Medical Opinion and Movement

**Published:** 1906-12-29

**Authors:** 


### Medical Opinion and Movement.

There is good reason to believe that the influenza bacillus is one of these air-pervading microbes and that infection takes place in the manner already indicated, rather than directly from one person to another. In this case the impure air caused by large gatherings of people in badly ventilated public places would easily lead to a toxic concentration of the microbe in the air inhaled. The problem of providing fresh air for a large number of people in a confined space without producing an unpleasant draught has yet to be solved, but in the meantime it is difficult to convince people that reinhaled stagnant air is far more injurious in its effects than the dreaded draught. In cannot be gainsaid that draughts are not wholly innocuous, but susceptibility to a cold current of air is largely a matter of custom and can be overcome. On the other hand, the spread of microbic infection probably takes place rather through the accumulation of the organisms in the stagnant air of a public conveyance, a public gathering, or the home itself, than by direct contagion from one individual to another. It has even been suggested that the prevalence of influenza in winter may be accounted for by the fact that people then congregate in greater numbers in buildings and exclude air more rigorously in their dwellings.

With the influenza bacillus making havoc in our midst, once more prostrating the high and the low of the land, the lay Press has been very busy lately discussing the causes and conditions which determine these annual winter visitations of the unwelcome intruder. In spite of our knowledge of micro-organisms and the causation of infectious diseases by them, the actual modes of transmission of infection are in many cases not yet fully explained. Laboratory experiments teach us that the incidence of any disease depends largely upon the actual dose of the specific microbe, and it has been suggested that at least certain pathogenic micro-organisms tend to drift through the atmosphere in swarms or clouds, setting up the disease in those with whom this cloud happens to collide. Those who are already victims of the disease probably form a sort of microbic halo around their persons, which varies in density and therefore in virulence for infection both to themselves and to others, according to the stagnation of [illegible]e surrounding atmosphere. It is on these grounds that the medical profession has been so insistent on the necessity of fresh air as a measure both of treatment and of prophylaxis for infectious diseases, and has for some time taught the importance of thorough ventilation in the house and in all public places, and endeavoured to eradicate the fallacies so prevalent in regard to these matters.

Morbid conditions of the stomach and their treatment have of late years absorbed a considerable amount of attention on the part of physicians and surgeons alike, largely on account of their constituting one of those borderland provinces in which the spheres of the physician and surgeon now more or less overlap. It is not surprising, therefore, that the discussion opened by Dr. Hale White and Mr. Mayo Robson at the Royal Medical and Chirurgical Society on the operative treatment of non-malignant ulcer of the stomach and its complications, aroused so much interest that it was necessary to adjourn the debate on two successive occasions. In regard to the treatment of simple gastric ulcer it was generally agreed that if after a trial of at least six months' consecutive medical treatment on the usual lines of rest and careful dieting, the trouble still persists, or a relapse occurs, operation should be resorted to, and the operation now recognised as the one *par excellence* for this and other allied gastric conditions is gastroenterostomy. Mr. Mayo Robson puts the mortality for this operation as low as 2 per cent. While statistics from other quarters, notably those supplied from Guy's Hospital, are not so favourable, it is generally agreed that in skilled hands the risks of the operation are not great. Perforation of course calls for immediate operation, as at least 95 per cent. of these cases are fatal unless operated upon. The condition of acute hæmorrhage is rarely fatal, whereas the mortality from operation during the hæmorrhage is 60 to 70 per cent.

The erection of sanatoria for the treatment of consumption, which is now taking place in all parts of the country, raises an important question in regard to the interests of the inhabitants of the district in which it may be proposed to establish such an institution, and the right they have to effectually protest against its being placed in their midst, if they unanimously object, or if they can show that it is likely to have an injurious effect upon the community. Such a case has arisen lately in connection with a consumption sanatorium established at Eldwick. This sanatorium consists of a number of houses in the centre of the village which have been purchased by a company for the purpose. A public meeting of the residents has been held to protest against its continuance on the grounds that owing to insufficient land to the buildings, the patients have to use the lanes and roads of the village for recreation, and that they form a menace to the health of the village, and that the sanatorium has caused severe depreciation in the sales of the property around. A member of the District Council pointed out that they had some difficulty in doing anything in the matter unless they could legally show that the sanatorium was a nuisance to the neighbourhood. It has now been so thoroughly established that consumption is an infectious disease, that it is only reasonable to insist that a sanatorium, which necessarily forms a focus of infection, in spite of all precautions in regard to disinfection and destruction of sputum, etc., should be properly isolated from the rest of the community by a certain area of unoccupied land around, in which the patients can take their recreation without mixing with the inhabitants of the district; and the inhabitants should have the right to prevent buildings closely adjoining their dwellings being used for sanatorial purposes.

